# Surveillance to Track Progress Toward Polio Eradication — Worldwide, 2020–2021

**DOI:** 10.15585/mmwr.mm7115a2

**Published:** 2022-04-15

**Authors:** Amanda L. Wilkinson, Ousmane M. Diop, Jaume Jorba, Tracie Gardner, Cynthia J. Snider, Jamal Ahmed

**Affiliations:** ^1^Global Immunization Division, Center for Global Health, CDC; ^2^Polio Eradication, Director General’s Office, World Health Organization, Geneva Switzerland; ^3^Division of Viral Diseases, National Center for Immunization and Respiratory Diseases, CDC.

Since the Global Polio Eradication Initiative (GPEI) was established in 1988, the number of reported poliomyelitis cases worldwide has declined by approximately 99.99%. By the end of 2021, wild poliovirus (WPV) remained endemic in only two countries (Pakistan and Afghanistan). However, a WPV type 1 (WPV1) case with paralysis onset in 2021, was reported by Malawi a year after the World Health Organization (WHO) African Region (AFR) was certified as WPV-free and circulating vaccine-derived poliovirus (cVDPV) cases were reported from 31 countries during 2020–2021 (*1*,*2*). cVDPVs are oral poliovirus vaccine-derived viruses that can emerge after prolonged circulation in populations with low immunity and cause paralysis. The primary means of detecting poliovirus transmission is through surveillance for acute flaccid paralysis (AFP) among persons aged <15 years, with confirmation through stool specimen testing by WHO-accredited laboratories, supplemented by systematic sampling of sewage and testing for the presence of poliovirus (environmental surveillance). The COVID-19 pandemic caused disruptions in polio vaccination and surveillance activities across WHO regions in 2020; during January–September 2020, the number of reported cases of AFP declined and the interval between stool collection and receipt by laboratories increased compared with the same period in 2019 (*3*). This report summarizes surveillance performance indicators for 2020 and 2021 in 43 priority countries* and updates previous reports (*4*). In 2021, a total of 32 (74%) priority countries^†^ met two key surveillance performance indicator targets nationally, an improvement from 2020 when only 23 (53%) met both targets; however, substantial national and subnational gaps persist. High-performing poliovirus surveillance is critical to tracking poliovirus transmission. Frequent monitoring of surveillance indicators could help identify gaps, guide improvements, and enhance the overall sensitivity and timelines of poliovirus detection to successfully achieve polio eradication.

## Acute Flaccid Paralysis Surveillance

Two key performance indicators used to assess AFP surveillance quality are 1) the nonpolio AFP (NPAFP) rate,^§^ with a NPAFP rate of ≥2 per 100,000 persons aged <15 years considered sufficiently sensitive to detect circulating poliovirus, and 2) the collection of adequate stool specimens^¶^ from AFP patients, with a target of ≥80% stool specimen adequacy, which indicates that surveillance can effectively identify poliovirus among AFP patients. Surveillance indicators for 43 priority countries experiencing or at high risk for poliovirus transmission were reviewed (Table 1).

**TABLE 1:** National and subnational acute flaccid paralysis surveillance performance indicators, number of confirmed wild poliovirus, and circulating vaccine-derived poliovirus cases, by country — 43 priority countries, World Health Organization African, Eastern Mediterranean, European, South-East Asia, and Western Pacific regions, 2020–2021*

Year/WHO region/Country	No. of AFP cases (all ages)	Regional or national NPAFP rate^†^	Percentage				No. of confirmed cases	
			Subnational areas with NPAFP rate ≥2^§^	Regional or national AFP cases with adequate specimens^¶^	Subnational areas with adequate specimens	Population living in areas meeting both indicators**	WPV	cVDPV^††^
**2020**								
**African Region**	**19,643**	**5.1**	**NA**	**85.6**	**NA**	**NA**	**—^§§^**	**551**
Angola	383	2.4	77.8	82.2	61.1	37.3	—	3
Benin	278	5.4	100	88.1	91.7	94.5	—	3
Burkina Faso	1,181	11.8	100	86.0	92.3	95.2	—	65
Cameroon	605	5.4	100	77.9	50.0	40.3	—	7
Central African Republic	222	9.8	100	65.3	28.6	28.2	—	4
Chad	993	11.7	95.7	81.8	65.2	69.0	—	101
Congo	93	3.7	75.0	83.9	75.0	53.7	—	2
Côte d’Ivoire	742	6.0	100	85.0	36.4	30.9	—	64
Democratic Republic of the Congo	3,304	7.6	100	80.4	53.8	55.9	—	81
Equatorial Guinea	26	5.0	71.4	80.8	57.1	58.5	—	—**^§§^**
Ethiopia	1,343	2.9	90.9	86.8	90.9	93.3	—	36
Guinea	321	4.5	100	69.2	25.0	16.4	—	44
Guinea-Bissau	20	2.4	45.5	50.0	9.1	6.3	—	—
Kenya	336	1.6	29.8	86.3	68.1	17.4	—	—
Liberia	48	2.3	73.3	95.8	100	64.8	—	—
Madagascar	635	5.7	100	90.6	95.5	96.4	—	2
Malawi	134	1.4	25.0	88.8	75.0	12.5	—	—
Mali	376	3.4	90.9	76.1	45.5	59.9	—	52
Mauritania	17	0.9	26.7	64.7	13.3	0	—	—
Mozambique	375	2.6	72.7	78.7	63.6	38.1	—	—
Niger	585	4.7	100	71.8	25.0	24.1	—	10
Nigeria	6,324	7.0	100	94.6	100	100	—	8
Senegal	135	1.7	50.0	77.0	28.6	12.2	—	—
Sierra Leone	89	2.4	60.0	100	100	62.3	—	10
South Sudan	434	6.4	100	80.4	70.0	64.3	—	50
The Gambia	23	2.3	42.9	78.3	42.9	3.7	—	—
Togo	161	4.0	100	62.1	0	0	—	9
Uganda	460	2.1	46.7	90.2	86.7	46.6	—	—
**Eastern Mediterranean Region**	**20,336**	**9.8**	**NA**	**87.8**	**NA**	**NA**	**140**	**547**
Afghanistan	3,979	22.9	100	92.4	97.1	98.4	56	308
Djibouti	5	1.7	16.7	100	33.3	4.6	—	—
Egypt	1,009	3.0	85.2	94.5	92.6	93.8	—	—
Iran	618	3.2	87.1	98.5	100	85.6	—	—
Iraq	476	2.9	84.2	93.3	94.7	89.0	—	—
Pakistan	11,972	16.4	100	85.3	100	100	84	135
Somalia	376	4.8	90.5	94.7	95.2	96.6	—	14
Sudan	733	3.9	100	92.8	94.4	93.6	—	59
Syria	343	5.3	92.9	84.5	78.6	63.6	—	—
Yemen	825	6.8	95.7	77.1	52.2	43.6	—	31
**European Region**	**158**	**1.5**	**NA**	**92.4**	**NA**	**NA**	**—**	**1**
Tajikistan	83	2.4	60.0	92.8	100	18.1	—	1
Ukraine	75	1.0	24.0	94.5	76.0	19.1	—	—
**South-East Asia Region**	186	1.3	**NA**	86.0	**NA**	**NA**	**—**	**—**
Burma (Myanmar)^¶¶^	186	1.3	22.2	86.0	72.2	9.0	—	—
**Western Pacific Region**	**965**	**2.6**	**NA**	**62.9**	**NA**	**NA**	**—**	**1**
Papua New Guinea	65	1.9	31.8	53.8	27.3	0	—	—
Philippines	900	2.7	58.8	63.6	35.3	15.7	—	1
**2021**								
**African Region**	**24,250**	**6.2**	**NA**	**88.8**	**NA**	**NA**	**1**	**538**
Angola	470	3.0	88.9	82.3	66.7	46.7	—	—
Benin	259	4.9	100	88.4	91.7	97.0	—	3
Burkina Faso	1,400	14.5	100	90.2	100	100	—	2
Cameroon	755	6.7	100	82.9	50.0	43.7	—	3
Central African Republic	202	8.9	100	76.7	28.6	35.1	—	—
Chad	1,055	13.6	100	84.6	69.6	70.3	—	—
Congo	178	6.9	100	79.2	58.3	31.9	—	2
Côte d’Ivoire	738	6.6	100	85.0	75.8	81.9	—	—
Democratic Republic of the Congo	3,439	7.9	100	85.3	84.6	91.0	—	28
Equatorial Guinea	15	2.8	42.9	93.3	71.4	38.8	—	—
Ethiopia	1,694	3.7	90.9	91.5	100	94.5	—	10
Guinea	370	6.2	100	79.5	50.0	49.6	—	6
Guinea-Bissau	20	1.9	36.4	65.0	27.3	28.3	—	3
Kenya	657	3.0	78.7	86.3	66.0	52.1	—	—
Liberia	131	6.0	100	99.2	100	100	—	3
Madagascar	602	5.2	100	94.7	100	100	—	13
Malawi	177	1.9	50.0	75.1	50.0	54.8	1	—
Mali	448	4.6	100	84.6	81.8	80.7	—	—
Mauritania	122	6.4	100	86.1	73.3	81.2	—	—
Mozambique	467	3.1	100	73.9	27.3	19.2	—	2
Niger	627	4.9	100	83.6	75.0	75.0	—	17
Nigeria	7,790	8.0	100	93.9	100	100	—	415
Senegal	359	4.5	100	83.6	71.4	77.5	—	17
Sierra Leone	173	5.0	100	85.0	60.0	59.4	—	5
South Sudan	543	8.8	100	89.0	90.0	84.0	—	9
The Gambia	32	3.1	57.1	90.6	57.1	56.2	—	—
Togo	298	8.6	100	91.6	100	100	—	—
Uganda	1,229	5.4	100	90.6	100	100	—	—
**Eastern Mediterranean Region**	**22,166**	**10.9**	**NA**	**87.8**	**NA**	**NA**	**5**	**71**
Afghanistan	4,095	25.5	100	93.4	100	100	4	43
Djibouti	8	2.7	16.7	75.0	0	0	—	—
Egypt	1,251	3.6	100	90.9	88.9	89.4	—	—
Iran	681	3.5	100	97.5	100	100	—	—
Iraq	709	4.2	94.7	91.1	94.7	85.5	—	—
Pakistan	13,084	18.0	100	85.0	100	100	1	8
Somalia	349	4.6	85.7	96.0	95.2	83.0	—	1
Sudan	637	3.6	100	94.0	100	100	—	—
Syria	431	6.7	92.9	85.4	78.6	61.9	—	—
Yemen	921	7.5	100	81.7	78.3	67.0	—	19
**European Region**	**294**	**2.4**	**NA**	**91.8**	**NA**	**NA**	**—**	**34**
Tajikistan	178	4.1	100	87.1	80.0	99.7	—	32
Ukraine	116	1.5	32.0	99.1	80.0	35.8	—	2
**South-East Asia Region**	**33**	**0.2**	**NA**	**84.8**	**NA**	**NA**	**—**	**—**
Burma (Myanmar)^¶¶^	33	0.2	0	84.8	33.3	0	—	—
**Western Pacific Region**	**975**	**2.6**	**NA**	**74.6**	**NA**	**NA**	**—**	**—**
Papua New Guinea	52	1.3	27.3	50.0	18.2	0	—	—
Philippines	923	2.7	11.8	76.6	47.1	20.5	—	—

**Abbreviations:** AFP = acute flaccid paralysis; cVDPV = circulating vaccine-derived poliovirus; NA = not applicable; NPAFP = nonpolio acute flaccid paralysis; WHO = World Health Organization; WPV = wild poliovirus.

* Data as of March 25, 2022.

^†^ Per 100,000 persons aged <15 years per year.

^§^ For all subnational areas regardless of population size.

^¶^ Standard WHO target is adequate stool specimen collection from ≥80% of AFP cases, assessed by timeliness and condition. For this analysis, timeliness was defined as two specimens collected ≥24 hours apart (≥1 calendar day in this data set), both within 14 days of paralysis onset. Good condition was defined as arrival of specimens in a WHO-accredited laboratory with reverse cold chain maintained and without leakage or desiccation.

** Percentage of the country’s population living in subnational areas that met both surveillance indicators (NPAFP rates ≥2 per 100,000 persons aged <15 years per year and ≥80% of AFP cases with adequate specimens).

^††^ Includes both cVDPV1 and cVDPV2; cVDPV was associated with ≥1 case of AFP with evidence of community transmission and genetically linked. https://polioeradication.org/wp-content/uploads/2016/09/Reporting-and-Classification-of-VDPVs_Aug2016_EN.pdf

^§§^ Dashes indicate that no confirmed cases were found.

^¶¶^ *MMWR* uses the U.S. Department of State’s short-form name “Burma”; WHO uses “Myanmar.”

**African Region.** Among 28 priority countries in AFR, 50% met both national surveillance indicator targets in 2020 and 79% met the targets in 2021 (as of March 25, 2022). Subnational surveillance performance also improved in AFR; both surveillance indicator targets were met in 52% of first subnational administrative level areas in 2020 and 75% in 2021 (Figure). In AFR, cVDPV type 2 (cVDPV2) cases were reported from 22 countries during 2020–2021; among 525 cVDPV2 cases reported in 2021, a total of 415 (79%) were from Nigeria. One WPV1 case was detected in a child in Malawi with paralysis onset in 2021 (*5*), approximately 1 year after AFR was certified as WPV-free; this is the first WPV1 case reported in AFR since 2016 and the isolate is genetically linked to a WPV1 lineage last detected in Pakistan in 2019.

**FIGURE:**
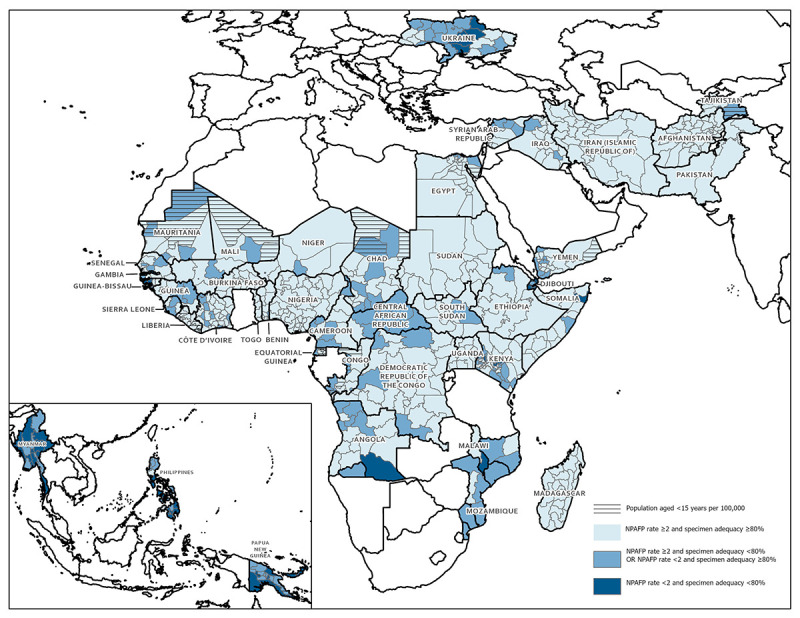
Combined performance indicators for the quality of acute flaccid paralysis surveillance* in subnational areas of 43 priority countries — World Health Organization African, Eastern Mediterranean, South-East Asia, and Western Pacific regions, 2021 **Abbreviations**: AFP = acute flaccid paralysis; NPAFP = nonpolio acute flaccid paralysis; WHO = World Health Organization. * Targets: ≥2 NPAFP cases per 100,000 persons aged <15 years per year and ≥80% of persons with AFP having two stool specimens collected ≥24 hours apart and within 14 days of paralysis onset, and arrival at a WHO-accredited laboratory by reverse cold chain (storing and transporting samples at recommended temperatures from the point of collection to the laboratory) and in good condition (i.e., without leakage or desiccation).

**Eastern Mediterranean Region.** Among 10 priority countries in the WHO Eastern Mediterranean Region (EMR), eight met both national surveillance indicator targets in 2020 and all but one (Djibouti with stool adequacy of 75%) met both targets in 2021. Most EMR countries performed well at the subnational level, but gaps were apparent in Djibouti. In 2020, a total of 140 WPV1 cases were detected in EMR countries (56 in Afghanistan and 84 in Pakistan), compared with five in 2021 (four in Afghanistan and one in Pakistan). Cases of cVDPV2 in EMR countries declined from 516 in 2020 to 68 in 2021, and cVDPV1 cases declined from 31 in 2020 to three in 2021 (all from Yemen).

**European Region.** In the WHO European Region (EUR), surveillance performance was assessed in Tajikistan and Ukraine. In 2020 and 2021, Tajikistan met both national indicators, whereas Ukraine met only the stool adequacy target. In Tajikistan, the proportion of the population living in areas that met both indicators increased significantly from 2020 to 2021.

**South-East Asia Region.** Surveillance performance was assessed in the WHO South-East Asia Region (SEAR), country of Burma (Myanmar),** which met the national stool adequacy target (86.0% and 84.8%, respectively) in both 2020 and 2021, but not the NPAFP rate target (1.3 and 0.2 per 100,000 persons aged <15 years, respectively). Subnational surveillance performance was poor in both years and none of the subnational areas met both surveillance indicator targets in 2021.

**Western Pacific Region.** In the WHO Western Pacific Region (WPR), surveillance performance was assessed in Papua New Guinea and the Philippines. In 2020 and 2021, the Philippines met the NPAFP rate indicator, and Papua New Guinea did not meet either of the surveillance indicators. None of the subnational areas in Papua New Guinea met the indicator targets in either year; in the Philippines, 20.5% of the population lived in subnational areas in which both surveillance indicators were met in 2021 (Figure). One cVDPV2 case was reported from the Philippines in 2020, but none in 2021.

Genomic sequence analysis identified 43 cVDPV emergence groups globally in active transmission from AFP cases during 2020–2021. These included 30 cVDPV2 and four cVDPV1 emergences in 27 countries in 2020 and 24 cVDPV emergence groups (20 cVDPV2 and 4 cVDPV1) in 22 countries in 2021.

## Environmental Surveillance

Poliovirus environmental surveillance is the systematic collection and testing of sewage specimens to identify poliovirus circulation. Because paralysis occurs in <1% of poliovirus infections, environmental surveillance can detect poliovirus circulation even in the absence of confirmed paralytic polio cases (*6*). During 2020–2021, cVDPV2 was detected by environmental surveillance before identification of a confirmed AFP case in Afghanistan, Liberia, and Senegal, and by environmental surveillance only in Djibouti, Egypt, Gambia, Iran, Mauritania, and Uganda.

In Nigeria, the number of cVDPV2-positive environmental surveillance samples increased from five samples collected from two sites in 2020 to 299 samples collected from 77 sites in 2021. In Afghanistan and Pakistan, the number of cVDPV2-positive samples declined from 310 across 65 sites in 2020 (56% from Afghanistan) to 75 across 30 sites in 2021 (53% from Afghanistan). During 2020–2021, 27 cVDPV emergence groups (24 cVDPV2 and three cVDPV1) were detected in sewage samples collected in 32 countries, including 22 (69%) from AFR, seven (22%) from EMR, two (6%) from WPR, and one (3%) from EUR.

In Afghanistan, WPV1 was isolated from only one environmental surveillance sample in 2021 compared with 35 samples from 15 sites in 2020 (*7*). In Pakistan, WPV1-positive samples also declined from 434 across 67 sites in 2020 to 65 across 34 sites in 2021 (*8*).

## Global Polio Laboratory Network

The WHO Global Polio Laboratory Network (GPLN) comprises 145 quality-assured poliovirus laboratories in the six WHO regions. GPLN laboratories implement standardized protocols to 1) isolate polioviruses (all laboratories); 2) conduct intratypic differentiation (ITD) to distinguish between WPV, Sabin (oral poliovirus vaccine) virus, and VDPV (134 laboratories); and 3) conduct genomic sequencing (28 laboratories). Poliovirus transmission pathways are monitored through sequence analysis of the viral protein 1 (VP1) capsid protein from virus isolates. The accuracy and quality of testing at GPLN laboratories are monitored through a comprehensive standardized quality assurance program of onsite reviews and proficiency testing (*9*). A different accreditation checklist with separate timeliness indicators is used for laboratories that conduct environmental surveillance.

GPLN tested 147,582 stool specimens in 2020 and 170,881 in 2021 (Table 2); cVDPVs were isolated from 1,067 AFP cases in 2020 and from 659 in 2021. From 2020 to 2021, the number of cVDPV isolates decreased from 530 to 521 in AFR, from 533 to 70 in EMR, and from two to zero in WPR; the number increased from two to 68 in EUR and was zero for both years in SEAR. During both 2020 and 2021, GPLN laboratories in all regions met the overall timeliness for onset to ITD results (80% of specimens within 60 days), and all but EUR in 2021 met the timeliness indicators for poliovirus isolation (80% of specimens within 14 days), 79% on time.

**TABLE 2:** Number of poliovirus isolates from stool specimens of persons with acute flaccid paralysis and timing of results, by World Health Organization region — worldwide, 2020 and 2021*

WHO region/Year	No. of specimens	No. of poliovirus isolates			% Poliovirus isolation results on time**	% ITD results within 7 days of receipt at laboratory^††^	% ITD results within 60 days of paralysis onset
		Wild^†^	Sabin^§^	cVDPV^¶^			
**African Region**							
2020	47,914	0	3,314	530	91	91	80
2021	58,004	1	3,396	521	89	79	85
**American Region**							
2020	1,066	0	12	0	81	82	82
2021	1,152	0	6	0	83	100	100
**Eastern Mediterranean Region**							
2020	40,179	245^§§^	1,311	533	96	61	95
2021	43,370	5	1,050	70	97	97	94
**European Region**							
2020	2,016	0	24	2	89	73	82
2021	2,350	0	53	68	79	96	95
**South-East Asia Region**							
2020	44,799	0	1,315	0	94	95	90
2021	53,649	0	1,030	0	93	89	90
**Western Pacific Region**							
2020	11,608	0	124	2	96	100	84
2021	12,356	0	58	0	97	100	99
**Total** ^¶¶^							
**2020**	**147,582**	**245**	**6,100**	**1,067**	**94**	**84**	**92**
**2021**	**170,881**	**6**	**5,593**	**659**	**93**	**84**	**88**

**Abbreviations**: AFP = acute flaccid paralysis; cVDPV = circulating vaccine-derived poliovirus; ITD = intratypic differentiation; VDPV = vaccine-derived poliovirus; WHO = World Health Organization; VP1 = viral protein 1; WPV = wild poliovirus.

* Data as of March 31, 2022.

^†^ Number of AFP cases with WPV isolates.

^§^ Either 1) concordant Sabin-like results in ITD test and VDPV screening, or 2) ≤1% VP1 nucleotide sequence difference compared with Sabin vaccine virus (≤0.6% for type 2).

^¶^ Includes both cVDPV1 and cVDPV2. For cVDPV types 1 and 3, ≥10 VP1 nucleotide differences from the respective poliovirus; for cVDPV2, ≥6 VP1 nucleotide differences from Sabin type 2 poliovirus.

** Results reported within 14 days of receipt of specimen.

^††^ Results of ITD reported within 7 days of receipt of specimen.

^§§^ Number of specimens with WPV isolates.

^¶¶^ For the last three indicators, total represents weighted mean percentage of regional performance.

Since 2017, the WPV1 South Asia genotype is the only WPV1 genotype that has been detected globally. Orphan isolates (isolates with ≤98.5% genetic identity in the VP1 capsid region, compared with other isolates) accounted for 18 of 140 (13%) WPV1 isolates from AFP patients in 2020 and two of six (33%) in 2021.

## Discussion

All priority countries faced setbacks in surveillance performance in 2020 because of the COVID-19 pandemic and associated risk mitigation measures (*3*); in 2021, AFP surveillance performance indicators rebounded in many countries. Several AFR countries’ subnational performance on surveillance indicators in 2021 improved compared with their prepandemic performance in 2019, including Burkina Faso, Côte d’Ivoire, Democratic Republic of the Congo, and Niger (*4*). Subnational surveillance gaps were apparent among one or more priority countries in each WHO region that included a priority country. Although WPV1 cases significantly declined in 2021, the recent detection of a WPV1 case in Malawi demonstrates that all countries remain at risk for WPV1 until global transmission is interrupted and underscores the critical importance of maintaining sensitive poliovirus surveillance in all countries, even those considered to be at low risk. An updated Global Polio Surveillance Action Plan for 2022–2024 was developed to guide and monitor surveillance system improvements at all levels of the GPEI (*10*); the plan is applicable globally but focuses on 30 priority countries.

The findings in this report are subject to at least three limitations. First, issues related to security and hard-to-access populations could affect AFP surveillance and limit interpretation of surveillance indicators. Second, high NPAFP rates do not necessarily indicate highly sensitive surveillance because not all reported AFP cases might meet the case definition, some actual AFP cases might go undetected, and background NPAFP rates might vary. Finally, the accuracy of stool specimen adequacy depends on whether the field investigator can elicit an accurate paralysis onset date.

High-quality surveillance is critical to reaching the milestone of global polio eradication and includes timely and effective AFP case detection, notification, and investigation; specimen transport; and laboratory testing. Frequent monitoring of surveillance indicators could help identify gaps, guide improvements, and enhance the overall sensitivity and timelines of poliovirus detection to successfully achieve polio eradication.

**Summary**

**What is already known about this topic**

Acute flaccid paralysis (AFP) surveillance, the primary means of tracking poliovirus transmission, is supplemented by environmental surveillance of sewage samples. The COVID-19 pandemic negatively affected polio surveillance.

**What is added by this report?**

Analysis of 2020–2021 AFP surveillance data from 43 priority countries experiencing or at high risk for poliovirus transmission found that national AFP surveillance performance improved from 2020 to 2021 in many priority countries, particularly in the World Health Organization’s African Region; however, substantial national and subnational gaps persist.

**What are the implications for public health practice?**

Surveillance gaps need to be identified and addressed to ensure timely detection of poliovirus circulation and achieve eventual eradication.

## Footnotes

* Countries selected for this 2020–2021 report were identified as priority countries in the WHO Global Polio Surveillance Action Plan (GPSAP), 2022–2024 because of persistent surveillance gaps and susceptibility to poliovirus transmission or had ≥1 WPV1 or cVDPV isolates detected from AFP or environmental surveillance in 2021 (https://polioeradication.org/wp-content/uploads/2022/03/GPSAP_2022-2024.pdf ). Note: In 2022, VDPV3 was isolated from an AFP case and genetically linked to VDPV3 strains isolated from environmental samples collected in Israel and the Palestinian Territories in 2021–2022 (https://www.euro.who.int/en/countries/israel/news/news/2022/3/circulating-vaccine-derived-poliovirus-confirmed-in-israel); the emergence was confirmed as cVDPV3 in March 2022; surveillance performance in these geographies are not included in this report.

† 2021 priority countries (30 GPSAP priority countries indicated by [G]): *African Region****:*** Angola (G), Benin (G), Burkina Faso (G), Cameroon (G), Central African Republic (G), Chad (G), Congo, Côte d’Ivoire (G), Democratic Republic of the Congo (G), Equatorial Guinea (G), Ethiopia (G), Gambia, Guinea (G), Guinea-Bissau (G), Kenya (G), Liberia, Madagascar (G), Malawi, Mali (G), Mauritania, Mozambique (G), Niger (G), Nigeria (G), Senegal, Sierra Leone, South Sudan (G), Togo (G), and Uganda; *Eastern Mediterranean Region****:*** Afghanistan (G), Djibouti, Egypt, Iran, Iraq (G), Pakistan (G), Somalia (G), Sudan (G), Syria (G), and Yemen (G); *European Region****:*** Tajikistan and Ukraine; *South-East Asia Region:* Burma (Myanmar) (G); *Western Pacific Region*: Papua New Guinea (G) and the Philippines (G).

§ The number of NPAFP cases per 100,000 persons aged <15 years per year.

¶ Two stool specimens collected ≥24 hours apart and within 14 days of paralysis onset, and arrival at a WHO-accredited laboratory by reverse cold chain (storing and transporting samples at recommended temperatures from the point of collection to the laboratory) and in good condition (i.e., without leakage or desiccation).

** *MMWR* uses the U.S. Department of State’s short-form name “Burma”; WHO uses “Myanmar.”
